# Sociodemographic factors associated with HbA1c variability in type 2 diabetes: a prospective exploratory cohort study

**DOI:** 10.1186/s12902-020-00585-6

**Published:** 2020-07-08

**Authors:** Emelia Mellergård, Per Johnsson, Frida Eek

**Affiliations:** 1 Department of Health Sciences, Faculty of Medicine, Lund University, Box 157, 22100 Lund, Sweden; 2 Department of Psychology, Faculty of Social Sciences, Lund University, Lund, Sweden

**Keywords:** Type 2 diabetes, Glycemic control, HbA1c variability, Sociodemographic factors, Sex differences, BMI, Diabetes management

## Abstract

**Background:**

The associations between sociodemographic factors and HbA1c variability in type 2 diabetes are not yet established. Examining group differences in HbA1c variability may help identify patient characteristics related to diabetes management. The present study examined differences in baseline HbA1c and HbA1c variability between groups with regard to sex, level of education, civil status, age, and BMI, in a sample of individuals with type 2 diabetes.

**Methods:**

The study was a prospective exploratory cohort study. Differences in HbA1c variability between sociodemographic groups were analyzed in 158 individuals. HbA1c variability was assessed as the standard deviation (SD) and coefficient of variation (CV) over five measured points, and a questionnaire was used to assess sociodemographic factors.

**Results:**

The results showed significantly higher HbA1c variability in men compared to women (mean difference 1.44 mmol/mol [95% CI: 0.58 to 2.31]), and significantly higher HbA1c variability in individuals with a BMI characterized as obese compared to individuals with a BMI characterized as normal weight (mean difference 1.56 mmol/mol [95% CI: 0.25 to 2.88]). There were no significant associations between HbA1c variability and civil status or education.

**Conclusions:**

Men and individuals with obesity may be more vulnerable to future diabetic complications than other groups, since they have greater long-term glycemic variability.

## Background

In 2017, the worldwide estimated number of people with diabetes was 425 millions, and in the same year diabetes accounted for 10.7% of global all-cause mortality among people aged 20–79 years. Type 2 diabetes accounts for 90% of all cases of diabetes, and is most commonly found in older adults, although increasingly seen in children and younger adults [1]. Type 2 diabetes is characterized by chronically elevated blood glucose levels, due to inadequate production of insulin and an inability of the body to effectively respond to insulin. Today, type 2 diabetes is a leading cause of cardiovascular disorders, blindness, end-stage renal failure, amputations and hospitalizations, as well as associated with increased risk of other disabling or deadly conditions such as cancer, chronic liver disease and cognitive decline [2].

HbA1c reflects the average glucose level over the past 8–12 weeks, and is commonly used both in research and in clinical settings as a measure of glycemic control. Attaining an ideal HbA1c level demonstrates control over the disease and enables prevention of its complications [3]. Target levels for HbA1c in type 2 diabetes are individual, but a general aim stated by the National Institute for Health and Excellence is 6.5–7.0% (48–53 mmol/mol) [4]. In recent years there has been an increased focus on adverse outcomes associated with glycemic variability [5–7]. Glycemic variability can be a sign of excess glycemic excursions, and, consequently, a risk of hyperglycemia or hypoglycemia [8]. Greater HbA1c variability is associated with adverse outcomes in several micro- and macrovascular end-points, as well as with mortality [6, 9–11]. Long-term glycemic variability is usually based on serial measurement of HbA1c, which reveals a general pattern of glycemic control over time. Previous studies indicate that HbA1c variability could possibly be superior for predicting diabetes-related complications compared to mean HbA1c [12–14]. Hence, considering HbA1c variability in clinical risk assessment may be beneficial [6]. It has been suggested that the association between HbA1c variability and diabetic complications may be explained by confounding factors, such as poor self-management or lack of support [13, 15]. Recent guidelines from both the American Diabetes Association and the European Association for the Study of Diabetes acknowledge the need for personalized care regarding both lifestyle advice and pharmacological treatment, recognizing that the experience of diabetes differs across roles, phase of the disease, the patient’s stage in life, accumulated experience of the disease over time, and a person’s cultural, political, economic and social context [16]. Level of education reflects one aspect of an individual’s socioeconomic position, and is presumed to influence access to and quality of care, diabetes-related knowledge and ability to adhere to treatment regimen by turning information into health enhancing behaviors [17, 18]. Spousal support may affect weight status and diabetes control, but the evidence is mixed and unclear [19]. Sex and age are both important factors influencing health, either independently or as covariates [16]. Excess bodyweight, as indicated by increased BMI, is an important risk factor for both mortality and numerous health issues [20]. However, the associations between sociodemographic factors and HbA1c variability are less well explored compared to other diabetes risk factors, and not as extensively studied in longitudinal studies [3]. Studying group differences in HbA1c variability based on these factors, may help identify patient characteristics related to long-term diabetes management, and enable a more focused approach to health care support of patients with type 2 diabetes. The aim of the present study was to examine differences in glycemic control related to sociodemographic factors. More specifically, the study aimed at exploring differences regarding baseline level HbA1c as well as HbA1c variability over 24 months, between different groups with respect to sex, level of education, civil status, age, and BMI. A further objective was to examine whether any potential effect on HbA1c variability was modified by sex.

## Methods

### Study setting

The present study was carried out as part of the study “Detailed Assessment of Type 2 diabetes” (DIACT). The primary aim of the DIACT study is to map different pathophysiological components relevant to type 2 diabetes progression, as well as investigate the role of lifestyle and psychological factors affecting disease management over time. In the present study, data on sociodemographic factors, glycemic control and diabetes severity over a time period of 24 months were analyzed. Participants were assessed at a diabetes daycare clinic at Skåne university hospital. Each individual had a baseline assessment and repeated measures at 6 months, 12 months, 18 months, and 24 months.

### Study sample

A selection of type 2 diabetes patients was made from the patient registry “All New Diabetics in Scania” (ANDIS), which is a combined research and quality assurance project that collects information about all new cases of diabetes in the region. Participants were recruited based on their type 2 diabetes diagnosis and age (35–75 years), and continuously enrolled in the study. Exclusion criteria were other endocrine disorders, pregnancy, GAD-antibodies, ongoing medication (e.g. cortisone) that could affect blood glucose levels, injury or disease that could affect measurement accuracy or challenge the individual’s health upon participation, inability to comprehend the implications of participation in the study, or participation in any other, ongoing study that could affect or be affected by the present study. Between May 2013 and September 2015, 1361 individuals (812 men and 549 women) were contacted for participation in the study. A first screening of eligibility of those interested in participating was made over the phone by research staff, and those eligible for participation moved on to a visit with a medical doctor for further screening. In the end, a total of 198 participants were included. The present study reports the results from participants enrolled in the study between May 2013 and March 2016, which accounts for 195 individuals. During the course of the study, 3 participants were excluded due to medical reasons, 2 participants were diseased before follow-up, 19 participants had less than five measurements of HbA1c, and 13 participants dropped out before follow-up. Hence, the final sample for the present study consisted of 158 participants.

### Measures

#### Sociodemographic factors

The participants responded to a questionnaire including questions about sociodemographic factors; level of education, civil status, age, and sex. Educational level was measured with the question “What is your highest level of education?”. The response alternatives were “elementary school”, “junior secondary school”, “2-year secondary high school”, “3-4-year secondary high school”, “university or college of higher learning, less than 3 years”, “university or college of higher learning, more than 3 years”, and “other education”. “Other education” (*n* = 12) was categorized as “2-year secondary high school” or “3–4-year secondary high school” based on participants’ reported occupation, which reflected their educational background. Marital status was based on five response categories; “married/cohabiting”, “single”, “divorced”, “living apart”, and “widow/widower”. Answers were categorized into two groups: In a relationship (“married/cohabiting”, “living apart”), and single (“single”, “divorced”, “widow/widower”). BMI was computed based on baseline assessment of height and weight, and categorized into three groups: normal weight (BMI = 18.50–24.99), overweight (BMI = 25.00–29.99) and obesity (BMI > 30). Age was assessed at baseline and categorized into three groups: < 65 years, 65–70 years, and > 70 years.

#### Clinical parameters

Blood samples were collected in EDTA tubes, and HbA1c was measured using Capillarys 3 TERA hemoglobin A1c Kit-program with CV of 3%. All measurements were performed at the same laboratory at Skåne University Hospital. HbA1c was assessed as a measurement of glycemic control and measured every 6 months in each participant. HbA1c variability was defined as the standard deviation (SD) over the five measure points, as well as the coefficient of variation (CV) calculated as (SD/mean HbA1c) *100 [18, 21]. Time since diagnosis was measured in years and treated as a continuous variable, and metformin treatment was based on participants reported use and dichotomized as yes or no.

### Procedure

Blood samples, body measurements and glucose measurements were obtained during the first visit. HbA1c was repeatedly measured at each visit. Laboratory analyses were carried out at local laboratories. The questionnaire data, assessing sociodemographic variables and the clinical parameters diabetes duration and drug treatment, was attained at the first visit.

### Statistical analysis

All statistical analyses were performed using IBM SPSS Statistics for Macintosh, Version 23.0. Descriptive statistics; percentage or means and SD, were computed for sex, civil status, BMI, education, metformin use, insulin use, years with diabetes, HbA1c at baseline (mmol/mol, %), and age. Group differences in baseline HbA1c based on age, sex, civil status, BMI, and education, were examined through a multivariable analysis of variance, including all sociodemographic variables as independent variables. Metformin treatment and diabetes duration were included as potential confounding variables. Differences regarding HbA1c-variability between groups based on age, sex, education, BMI, and civil status were also analyzed through a multivariable analysis of variance, including all sociodemographic variables as independent variables. Metformin treatment, disease duration, and baseline HbA1c were included in the model as potential confounders. Potential effect modifications were explored by including interaction effects between sex and BMI, and between sex and civil status, in the model. The analysis was performed stepwise, using backwards elimination of potential confounding variables in order to only adjust for potential confounding factors that significantly contributed to the variance of the dependent variable. Limit of significance was set to ≤0.05 in all analyses. Precision of estimates was assessed by 95% CI. Since the study was exploratory, rather than hypothesis driven, no a priori sample size calculation was made.

## Results

There were more men (65.2%) than women (34.8%) participating in the study. The mean age was 67.6 years, and mean time since diagnosis was 4.1 years. The mean baseline HbA1c was 6.4% (46.9 mmol/mol). A large proportion of the participants (72.1%) were prescribed metformin. Most participants had a BMI categorized as either overweight (48.4%) or obese (38.9%). Different education levels were represented in the sample, and roughly equally distributed on a high to low continuum (Table 1).

**Table 1:** Sample characteristics (*N* = 158)

Age (years)	
Mean (SD)	67.6 (7.0)
< 65 years	25.9 (41)
65–70 years	35.4 (56)
> 70 years	38.6 (61)
Sex	
Women	34.8 (55)
Men	65.2 (103)
BMI (*n* = 157)	
Normal weight (BMI ≤ 24.99)	12.7 (20)
Over weight (BMI = 25–29.99)	48.4 (76)
Obese (BMI ≥ 30)	38.9 (61)
Education level (*n* = 142)	
Elementary school	30.3 (43)
High school	40.1 (57)
University	29.6 (42)
Civil status (*n* = 147)	
In a relationship	81.6 (120)
Single	18.4 (27)
Years since diagnosis, mean (SD)	4.1 (3.4)
Baseline HbA1c, mean (SD)	
mmol/mol	46.9 (7.5)
%	6.4 (0.7)
Prescribed metformin	72.2 (114)
Prescribed insulin (*n* = 156)	7.1 (11)
Data are presented as percentage (*n*) unless otherwise indicated.	

There were no significant differences in HbA1c at baseline between groups based on civil status, sex, BMI, education, or age (Table 2). Further, neither diabetes duration nor metformin treatment significantly contributed to the variation in baseline HbA1c levels.

**Table 2:** Baseline HbA1c levels (mmol/mol, %) in different groups based on civil status, sex, BMI, education, and age

	HbA1c mmol/mol			HbA1c %		
	*Mean (95% CI)*	*Mean difference between groups (95% CI)*	*p*	*Mean (95% CI)*	*Mean difference between groups (95% CI)*	*p*
Civil status						
In a relationship (*n* = 116)	46.53 (44.82, 48.24)			6.41 (6.25, 6.57)		
Single (*n* = 26)	46.66 (43.61, 49.70)	−0.13 (−3.41, 3.16)	.938	6.42 (6.14, 6.70)	− 0.01(− 0.31, 0.29)	.938
Sex						
Men (*n* = 96)	46.28 (44.26, 48.30)			6.39 (6.20, 6.57)		
Women *(n* = 46)	46.90 (44.36, 49.44)	−0.62 (−3.35, 2.11)	.655	6.44 (6.21, 6.68)	−0.06 (− 0.31, 0.19)	.655
BMI						
Normal weight (*n* = 19)	44.73 (41.04, 48.42)			6.24 (5.91, 6.58)		
Overweight (*n* = 69)	46.66 (44.51, 48.82)	1.94^a^ (−2.02, 5.90)	.335	6.42 (6.22, 6.62)	0.18^a^(−0.19, 0.54)	.335
Obese (*n* = 54)	48.38 (46.10, 50.67)	3.66^a^ (−0.43, 7.75)	.079	6.58 (6.37, 6.79)	0.34^a^(−0.04, 0.71)	.079
Education						
Elementary school (*n* = 43)	46.29 (43.50, 49.08)			6.39 (6.13, 6.64)		
High school (*n* = 57)	46.20 (43.77, 48.63)	−0.09^b^ (−3.21, 3.03)	.954	6.38 (6.16, 6.60)	− 0.01^b^ (− 0.29, 0.28)	.954
University (*n* = 42)	47.28 (44.72, 49.85)	0.99^b^ (−2.33, 4.32)	.556	6.48 (6.24, 6.71)	0.09^b^ (−0.21, 0.40)	.556
Age						
< 65 years (*n* = 38)	46.72 (43.92, 49.51)			6.43 (6.17, 6.68)		
65–70 years (*n* = 51)	47.61 (45.08, 50.14)	0.90^c^ (−2.40, 4.20)	.592	6.51 (6.28, 6.74)	0.08^c^ (−0.22, 0.38)	.592
> 70 years (*n* = 53)	45.44 (42.96, 47.93)	−1.27^c^ (−4.59, 2.04)	.449	6.31 (6.08, 6.54)	−0.12^c^ (− 0.42, 0.19)	.449

^a^mean difference in relation to BMI group “Normal weight”

^b^mean difference in relation to Education group “Elementary school”

^c^mean difference in relation to Age group “< 65 years”

In HbA1c variability as absolute measure (SD), there were significant differences based on sex (*p* = .001) and BMI (*p* = .049). Men showed significantly greater HbA1c variability compared to women (mean difference 1.44 mmol/mol [95% CI: 0.58 to 2.31], *p* = .001). There was also a significant difference between individuals with a BMI characterized as normal weight and individuals with a BMI characterized as obesity (mean difference 1.56 mmol/mol [95% CI: 0.25 to 2.88], *p* = .020). Overall, HbA1c variability increased with greater BMI, and was lowest among individuals with a BMI characterized as normal weight (mean 2.54 [95% CI: 1.36 to 3.71]). Although not significant, the opposite was found regarding age; individuals in the oldest age-group (mean 2.84 [95% CI: 2.05 to 3.64]) had lower HbA1c variability compared to individuals in the youngest age-group (mean 3.65 [95% CI: 2.76 to 4.54]) and individuals who were 65–70 years old (mean 3.51 [95% CI: 2.71 to 4.31]). Finally, individuals in a relationship (mean 2.94 [95% CI: 2.40 to 3.49]) had lower HbA1c variability than those who were single (mean 3.72 [95% CI: 2.76 to 4.69]) (Table 3). There were no significant interaction effects between sex and BMI, or between sex and civil status.

**Table 3:** Multivariable analysis of variance of HbA1c (mmol/mol, %) variability in groups based on civil status, sex, BMI, education, and age

HbA1c variability mmol/mol				HbA1c variability %		
	*Mean (95% CI)*	*Mean difference between groups (95% CI)*	*p*	*Mean (95% CI)*	*Mean difference between groups (95% CI)*	*p*
Civil status						
In a relationship (*n* = 116)	2.94 (2.40, 3.49)			0.26 (0.21, 0.32)		
Single (*n* = 26)	3.72 (2.76, 4.69)	−0.78 (− 1.82, 0.26)	.142	0.34 (0.25, 0.43)	− 0.08 (− 0.18, 0.02)	.126
Sex						
Men (*n* = 96)	4.06 (3.41, 4.70)			0.37 (0.31, 0.43)		
Women (*n* = 46)	2.61 (1.81, 3.42)	1.44 (0.58, 2.31)	.001	0.24 (0.16, 0.31)	0.13 (0.05, 0.21)	.002
BMI						
Normal weight (*n* = 19)	2.54 (1.36, 3.71)			0.24 (0.13, 0.35)		
Overweight (*n* = 69)	3.37 (2.69, 4.06)	0.84^a^ (−0.43, 2.10)	.192	0.31 (0.24, 0.37)	0.07^a^ (−0.05, 0.19)	.244
Obese (*n* = 54)	4.10 (3.37, 4.83)	1.56^a^ (0.25, 2.88)	.020	0.36 (0.29, 0.43)	0.13^a^ (0.00, 0.25)	.005
Education						
Elementary school (*n* = 43)	3.40 (2.51, 4.28)			0.32 (0.23, 0.40)		
High school (*n* = 57)	3.44 (2.66, 4.21)	0.04^b^ (−0.95, 1.03)	.938	0.31 (0.23, 0.38)	0.01^b^ (−0.10, 0.08)	.827
University (*n* = 42)	3.17 (2.36, 3.98)	−0.23^b^ (−1.28, 0.83)	.673	0.28 (0.21, 0.36)	−0.03^b^ (− 0.13, 0.07)	.527
Age						
< 65 years (*n* = 38)	3.65 (2.76, 4.54)			0.34 (0.25, 0.42)		
65–70 years (*n* = 51)	3.51 (2.71, 4.31)	−0.14^c^ (−1.19, 0.91)	.789	0.30 (0.22, 0.38)	−0.04^c^ (− 0.14, 0.06)	.469
> 70 years (*n* = 53)	2.84 (2.05, 3.64)	−0.81^c^ (−1.86, 0.25)	.132	0.27 (0.20, 0.35)	−0.06^c^ (− 0.16, 0.04)	.209

^a^mean difference in relation to BMI group “Normal weight”

^b^mean difference in comparison to Education group “Elementary school”

^c^mean difference in comparison to Age group “< 65 years”

Results were similar when using relative HbA1c variability (CV) as outcome: significant differences between men and women (mean difference 2.41 mmol/mol [95% CI: 0.92 to 3.91], *p* = .002) were found, as well as a significant difference between individuals with a BMI characterized as normal weight and individuals with a BMI characterized as obesity (mean difference − 2.70 mmol/mol [95% CI: − 4.96 to − 0.44], *p* = .020) (data not shown). No significant differences between groups based on age, education or civil status were found for either HbA1c variability outcome measure.

## Discussion

The present study examined baseline HbA1c levels as well as differences in HbA1c variability over 2 years related to sociodemographic factors. The results showed significantly higher HbA1c variability in men compared to women, thus providing additional support that men tend to have less stable glycemic control compared to women [5]. In previous studies, sex and age have been found to be associated with hyperglycemia (HbA1c > 58 mmol/mol), and it has been suggested that different therapeutic goals for HbA1c in men and women might be an important consideration in diabetes care [22–24]. In a recent study, Noyes et al. (2017) found that men with type 2 diabetes had greater odds of having high HbA1c variability, and that both younger age and higher BMI were associated with higher HbA1c variability [5]. Older age and a higher frequency of HbA1c testing have in previous studies been associated with overall lower levels of HbA1c [25]. Sex differences in glycemic control probably have a multifactorial explanation, including both differences in biological mechanisms linked to diabetes, as well as differences in self-management, between men and women. Impairment in glucose and lipid metabolism, body composition, and energy metabolism, is to a high degree influenced by sex hormones, and factors influencing the development of obesity, such as health behaviors, income, and education, may differ between men and women [26]. Sex-specific differences regarding disease prevention and treatment are, while well studied in other fields of medicine, less well researched in metabolic and endocrine disorders [27]. Improving awareness of sex differences could help promote evidence-based, sex specific, clinical recommendations [28–30].

An unstable glycemic control could signal a lack of treatment adherence and patient compliance, and people with high HbA1c variability have previously been reported to have unhealthier lifestyle habits [31, 32]. In a systematic review and meta-analysis, Gorst et al. (2015) examined HbA1c variability and risk of adverse outcomes in diabetes, implicating HbA1c variability as an important factor in future clinical risk assessment [6]. In a recent review, Kovatchev (2017) also denotes the importance of a two-sided optimization of glycemic control; lowering absolute HbA1c levels, *and* keeping blood glucose variability stable [33]. An important aspect to discuss in light of the present findings is its clinical relevance, since to date, there is no clear consensus on the clinical interpretation of HbA1c variability. According to Hirakawa et al. (2014), the risk of major macrovascular events and all-cause mortality is significant only in individuals with HbA1c variability <− 0.3% or > 0.3% (<− 3.3 mmol/mol or > 3.3 mmol/mol) [11]. Forbes et al. (2018) suggests increments of 5.5 mmol/mol as an accepted indicator of clinical relevance in HbA1c variability [9]. In our sample, men and individuals with a BMI ≥ 30 had a HbA1c variability above the threshold proposed by Hirakawa et al. (2014), suggesting that these groups may be at a greater risk of developing diabetic complications. This is in line with previous studies that have found BMI and sex to be associated with higher HbA1c variability [5].

The results from the present study indicate that sex and BMI are factors that may affect HbA1c variability. In particular, men and individuals with obesity seem to have greater variability, indicating that these groups may be more vulnerable to future diabetic complications. Between 1980 and 2008, a global trend in increased BMI in both men and women was evident [20]. Ninety percent of patients with type 2 diabetes have a BMI greater than 23 kg/m^2^, and obesity and diabetes have common pathophysiology; impaired insulin production and action, impaired vascular function, and other metabolic anomalies [34, 35]. Diabetes management is an everyday, ongoing process, and a single measure of glycemic control might not capture the complexity of daily self-management efforts in order to maintain blood glucose levels in a healthy range. A simple continuum model proposed by Mulcahy et al. (2003) and adopted by the American Association of Diabetes Educators (AADE), suggests that successful diabetes management should be conceptualized as both learning, behavior change, clinical improvement and health status improvement [36]. Future studies that assess clinical improvement, including glycemic variability, as well as measurements of e.g. behavior, motivation and quality of life, could help explain the intricacy of successful long-term glycemic stability in a more comprehensive way.

The study sample in the present study had an overall good glycemic control and short diabetes duration, which may have implications for the generalizability of the results to individuals with less well-regulated diabetes and with longer disease duration [37]. Examining if similar differences in HbA1c variability can be found in groups of less well controlled individuals would supplement the findings of the present study. There is also no standardized method of measuring HbA1c variability, but the most common approach in previous research is to use the standard deviation or coefficient of variation of all HbA1c measurements in the period of investigation [38]. However, neither SD nor CV can be easily interpreted in clinical practice, making it difficult to evaluate the clinical impact of results where these measures are used [39]. Mehring, Donnachie & Schneider (2016) calls for consensus on a precise definition of HbA1c variability in order to advance research on long-term glycemic fluctuations and cardiovascular events [31]. Using the standard deviation as a measure of variability can be problematic if study participants have irregular follow-up intervals, with measures differently spaced. However, this was not an issue in the present study, since participants were assessed with an equal interval number of measurements. The results in the present study were similar using CV as a measure of HbA1c variability, indicating that the differences in SD were not merely a result of differences in mean HbA1c levels.

Of the participants in the present study, 65.2% were men and 34.8% were women. This distribution is approximately similar to the proportions found in ANDIS, the patient registry from which the participants were recruited (59.7% men and 40.3% women). Hence, the study sample could from a sex perspective be considered representative of the studied population. However, since the sample was not randomly selected it cannot be considered fully representative of the study population. This may limit the generalizability of the results. Further, potentially significant relationship between sociodemographic factors and the outcome may not have been fully disclosed due to possible lack of sufficient statistical power. A larger sample size would improve the reliability of the present results. Additional factors influencing diabetes management that have not been accounted for in the present study is another important consideration. Other medication, diet, and physical activity are possible confounding factors, affecting glycemic stability. Not including these factors in the analyses is a limitation of the present study. Future studies may also focus on psychological factors, such as patient competence, adjustment to diabetes, and motivation, as these are factors that could influence diabetes self-care and affect glycemic control over time.

## Conclusions

A focus on HbA1c variability, i.e. long-term fluctuation in HbA1c levels, has in recent years gained awareness, but the relations between sociodemographic factors and HbA1c variability are less well explored than other diabetes risk factors, and not as extensively studied in longitudinal studies. Exploring factors related to elevated HbA1c and greater HbA1c variability may indicate groups at increased risk of diabetic complications. In the present study, significant differences in HbA1c variability between men and women were found, as well as between groups with different BMI, suggesting that sex and BMI may need to be considered when optimizing glycemic control. Future studies will need to establish the clinical impact of these differences in glycemic control, as well as examine if the same results are found among individuals with less well-regulated HbA1c.

## Data Availability

The datasets used and analyzed during the current study are available upon reasonable request from the PI of the DIACT study. Requests can be sent to the corresponding author of the present study.

## Abbreviations

HbA1c: Glycosylated hemoglobin
BMI: Body mass index
SD: Standard deviation
CV: Coefficient of variation
